# Correlations between Demographic, Clinical, and Paraclinical Variables and Outcomes in Patients with KRAS-Mutant or KRAS Wild-Type Metastatic Colorectal Cancer—A Retrospective Study from a Tertiary-Level Center in Romania

**DOI:** 10.3390/diagnostics13182930

**Published:** 2023-09-13

**Authors:** Edvina Elena Pîrvu, Emilia Severin, Raluca Ileana Pătru, Irina Niță, Stefania Andreea Toma, Roxana Rodica Macarie, Cristina Elena Cocioabă, Ioana Florescu, Simona Coniac

**Affiliations:** 1 Department of Genetics, “Carol Davila” University of Medicine and Pharmacy, 050474 Bucharest, Romania; 2 Department of Medical Oncology, “Coltea” Clinical Hospital, 030167 Bucharest, Romania; 3 Department of Medical Oncology, Medicover Hospital, 020331 Bucharest, Romania; 4 Department of Medical Oncology, Ponderas Academic Hospital, 014142 Bucharest, Romania

**Keywords:** metastatic colorectal cancer, KRAS mutation, real-world data from Romania

## Abstract

Colorectal cancer (CRC) is a significant global public health concern and its characteristics in Eastern Europe are underexplored. In this retrospective study, data of 225 patients with metastatic colorectal cancer (mCRC) from the Colțea Clinical Hospital’s Oncology Department in Bucharest were analyzed between 2015 and 2023. They were divided into two groups based on the presence of KRAS mutation. The primary objective of the study was to investigate whether the presence of KRAS mutations influenced the prognosis of mCRC and to identify any demographic, clinical, or paraclinical factors associated with KRAS mutations in stage IV CRC. The overall survival for the entire study population was 29 months. There was a trend towards increased survival in the KRAS wild-type group (31 months) compared to the KRAS-mutant group (26 months), but this difference did not reach statistical significance. We found that lower levels of education, advanced T stage, advanced N stage, and M1 stage at diagnosis negatively impacted prognosis. Real-world data are crucial in shaping public policy strategies to better support patients with metastatic CRC. Understanding the correlations between the demographic, clinical, and paraclinical variables and the outcomes in mCRC patients with KRAS-mutant and KRAS wild-type colorectal cancer is essential for improving patient care and treatment strategies in Romania and beyond.

## 1. Introduction

With over 1.9 million new cases and 930,000 deaths estimated in 2020 according to GLOBOCAN and with a cancer burden that is projected to increase to 3.2 million new cases and 1.6 million deaths in the next 20 years, colorectal cancer (CRC) is a public health problem worldwide [1]. Although the incidence rates are higher in Western Europe due to lifestyle factors and also screening programs that allow early diagnosis, the mortality rates are higher in Eastern Europe (20.2 per 100,000 males) [2]. In Romania, an Eastern European country with a population of 19.27 million, in 2020, 98,886 cases were reported, with 13,000 new cases diagnosed [1]. The shift from a lower income economy to a higher income economy is linked with alterations in lifestyle that are likely to affect the occurrence of colorectal cancer (dietary factors, increased obesity incidence, and decreased physical activity). With no national screening program and a national cancer plan that is not yet functional, the burden of colorectal cancer will continue to be a challenge for the health system in Romania.

At diagnosis, between 15% and 30% of patients present with metastases, and up to 50% of patients with initially localized disease will develop metastases. The most common locations of metastases are the liver, lungs, peritoneum, and distant lymph nodes [3].

One of the most mutated oncogenes in CRC is Kirsten rat sarcoma (KRAS), with more than 40% of patients harboring activating KRAS missense mutations, most frequently in codons 12, 13, and 61 [4].

According to the European Society of Medical Oncology recommendations, KRAS status testing must be done in all patients diagnosed with metastatic colorectal cancer (mCRC) in order to select first-line therapy [3].

Given the scarcity of such research from Romania in the scientific literature, our study aims to explore possible correlations between demographic, clinical, and paraclinical variables and the outcomes in a metastatic KRAS-mutant and KRAS wild-type colorectal cancer population of 225 adult patients diagnosed with stage IV colorectal cancer and treated at the Coltea Clinical Hospital, Bucharest, Romania, a tertiary level center.

## 2. Materials and Methods

We performed an institutional review, ethically approved, retrospective, observational non-randomized study with a transversal section on a cohort of 225 adult patients diagnosed with stage IV colorectal adenocarcinoma treated at the Coltea Clinical Hospital, Bucharest, Romania, between 1 January 2015 and 1 February 2023. All the procedures in the study followed the ethical standards in the Helsinki Declaration. Our research was carried out with the approval and in accordance with the guidelines of the local Ethics Committee.

All the patients included in the study must have had a confirmed histopathological diagnosis of colorectal adenocarcinoma, a valid KRAS mutation test performed on solid biopsy, radiological confirmation of stage IV disease by computed tomography, and they must have received at least one line of systemic treatment in the metastatic setting. The exclusion criteria were the presence of a second primary at diagnosis, the presence of a brain metastasis at diagnosis, and the presence of NRAS or BRAF mutations. For the KRAS mutation testing, DNA was extracted from the sample under investigation (QIAmp DNA FFPE tissue kit, QIAmp DSP DNA Mini kit). A targeted resequencing assay (Ion AmpliSeq NGS Panel, Thermo Fisher Scientific, Romont, Fribourg, Switzerland) was used for mutation detection in exons 2, 3, and 4 of the KRAS genes. Sequencing was carried out using the next-generation sequencing platform Ion Gene Studio S5 Prime System (Thermo Fisher Scientific). The detection limit of the method is 2–5% of mutant allelic content, depending on the genomic region analyzed.

Based on their KRAS status, the patients were divided in two arms: Arm A included 88 patients with one or more KRAS mutations and Arm B included 137 patients with KRAS wild-type colorectal cancer.

The primary objective of this study was to determine if the presence of KRAS mutations influences the prognosis of metastatic colorectal cancer and to investigate if there are any demographic (age, sex, level of education, and rural versus urban), clinical (primary tumor localization, tumor grade (G), tumor stage (T), lymph node stage (N), metastatic disease at diagnosis, obesity, hypertension, diabetes, alcohol abuse of more than 3 units of alcohol per day, smoking history, and family history of colorectal cancer), or paraclinical particularities (carcinoembryonic antigen (CEA), carbohydrate antigen 19-9 (CA 19-9), lactate dehydrogenase (LDH), and alkaline phosphatase (ALK)) associated with KRAS mutations in stage IV colorectal cancer. The primary endpoints were overall survival (OS) and progression free survival (PFS).

For the levels of education, we applied the school stages used in Romania (i.e., Level I, primary and secondary school; Level II, high school; Level III, higher education including university and post-university studies). For the rural versus urban analysis, the patients were divided into two groups, i.e., urban or rural, taking into consideration each patient’s residence for the last 10 years. Clinical staging was performed according to the clinical TNM stage groups (tumor size, nodal status, and metastasis categories) at the time of diagnosis.

The secondary objective was to determine the influence of the clinical particularities on the prognosis of colorectal cancer patients. A classical descriptive statistical analysis of the variables included in the study was performed.

For continuous variables, central tendency was estimated with the mean and median, and variability tendency was estimated with standard deviation (SD), minimum, maximum, and the distribution range (the difference between maximum and minimum). The inferential analysis used methods like a time-to-event survival analysis. The methods used the follow statistical estimators:

- The Kaplan–Meier estimator, with graphical representation of the survival curves and calculations of the mean survival and restricted mean survival time (RMST) statistics, and for a more complete analysis, RMST statistics were compared on multiple moments of the survival curves (using the distribution quartiles of the follow-up period) at 17, 29, and 46 months;
- Hazard ratio (HR).

The level of significance for α in the study analysis was 0.05. Values smaller than 0.05 had statistical significance.

For the statistical analysis, the following software was used: software R, version 4.0.2, Copyright © 2020 The R Foundation for Statistical Computing, R Core Team (2020), R: A language and environment for statistical computing, R Foundation for Statistical Computing, Vienna, Austria, URL: https://www.R-project.org, accessed on 20 June 2023. The following supplementary packages were used: survival, survminer, survRM2, and gtsummary [5,6,7,8,9].

Similar to the majority of studies, the design of the current study was subject to limitations, the main one being the retrospective and the limited number of patients. Also, the 7-year duration of the study allowed the patients who were treated after 2020 to access new third-line therapies that were not accessible before, a factor that could have impacted survival. Another important limitation is the fact that mismatch repair deficiency/microsatellite instability and NRAS and BRAF testing were not reimbursed and not systematically tested in the clinic and their impact was not evaluated.

## 3. Results

Based on the inclusion and exclusion criteria, 225 patients were divided according to their KRAS status between Arm A (88 patients with one or more KRAS mutations) and Arm B (137 patients with KRAS wild-type colorectal cancer).

### 3.1. Demographic Characteristics

The comparative analysis of the demographic characteristics between the two arms is presented in Table 1.

The only difference between the two arms that reached statistical significance is the incidence of diabetes mellitus, with 18% of the patients in the KRAS wild-type group and only 8% of the patients in the KRAS-mutant group also diagnosed with diabetes. Although it was not statistically significant, a difference in the family history of cancer was observed, with 2.9% of the KRAS wild-type patients having at least one case of cancer in the family compared with 5.7% in the KRAS-mutant group.

### 3.2. Survival Analysis

**Figure 1:**
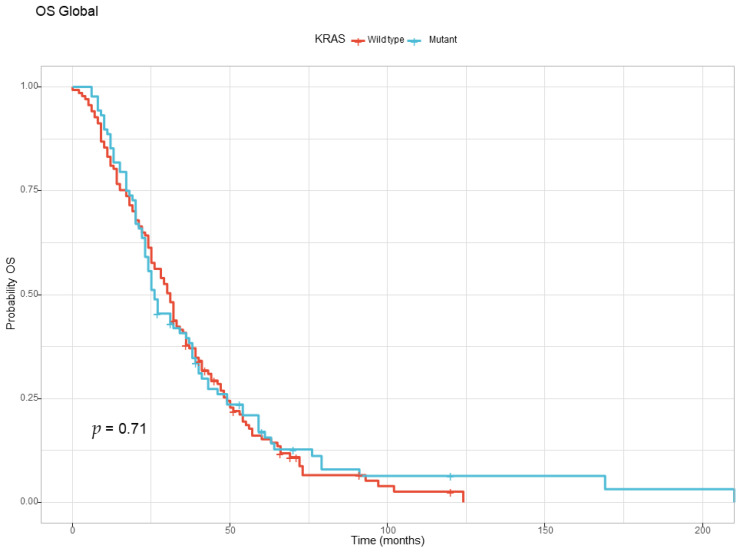
The Kaplan–Meyer curve for overall survival for the KRAS wild-type and KRAS-mutant groups.

The global overall survival for the entire study population was 29 months. By the end of the study, 206 out of 225 patients (91.55%) had succumbed to the disease. In the two groups, we noticed a tendency for increased survival in the KRAS wild-type group (31 months) compared with the KRAS-mutant group (26 months), but it did not reach statistical significance at the log-rank test. The global overall survival based on KRAS status is presented in Figure 1.

As presented in Table 2, the Cox regression could not find any differences with statistical significance between the KRAS wild-type and KRAS-mutant populations.

**Figure 2:**
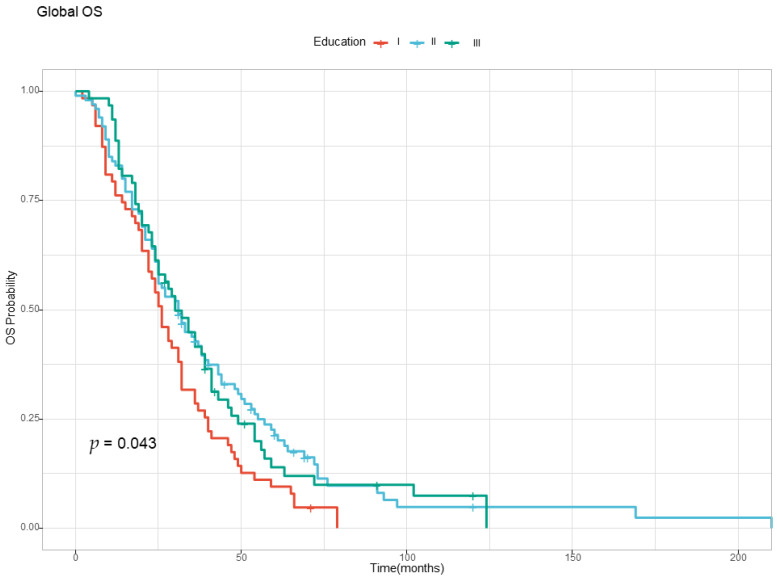
The Kaplan–Meyer curve for overall survival according to the level of education. Level I—Primary and secondary school; Level II—High school; Level III—University and post university studies.

The OS analysis found no differences based on sex, age, and urban versus rural background, but education levels influenced survival, as depicted in Figure 2. The patients were divided into three subgroups, based on their level of education (i.e., Level I—primary and secondary school, Level II—high school, Level III—university and post university studies). Patients with Level I education (primary and secondary school) had the worst prognosis, with a median OS of only 26 months (Figure 2 and Table 3).

Compared with patients with Level I education, for patients with Levels II and III education, the hazard ratio was 30% lower, the effects having a statistical significance.

As illustrated in Table 4 and Table 5, a statistically significant difference regarding T stage at diagnosis was confirmed, with T1 patients having the best prognosis. Compared with T1 patients, T4 patients had a hazard of death four times higher.

The OS analysis also found a difference that was statistically significant for N stage at diagnosis, with the N2 patients having the worst prognosis, as demonstrated in Table 6 and Table 7. Compared with the N0 patients, the N1 patients had a hazard of death 1.5 times higher and the N2 patients had a hazard of death 2.4 times higher, and both reached statistical significance.

**Figure 3:**
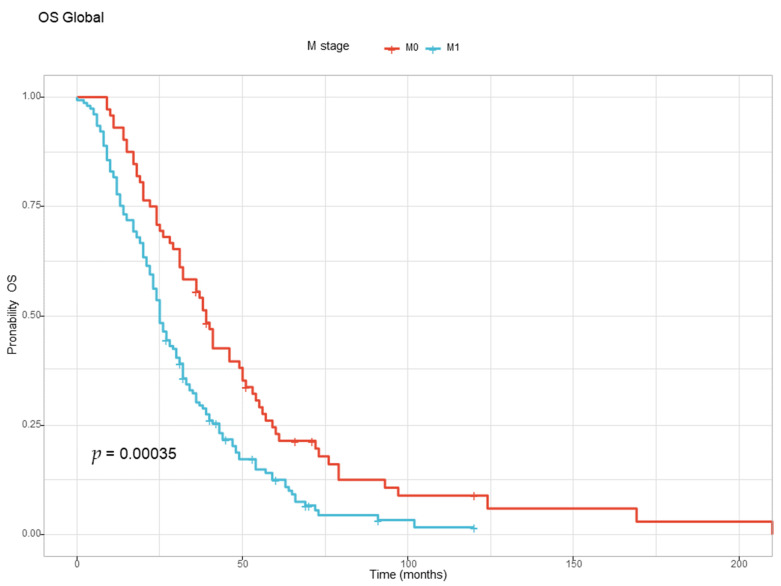
The Kaplan–Meyer curve for overall survival for the M1 and M0 groups at diagnosis.

The correlation between metastatic stage at diagnosis and OS was also analyzed. The patients with metastatic disease at diagnosis (M1) had a worse prognosis than the patients that were free of metastasis at diagnostic (M1) and developed metastatic disease later, as illustrated in Table 8 and Figure 3 below.

For the analysis of the location of the primary tumor, a multiple Cox model was used, knowing that our study did not consider synchronous tumors. The results are illustrated in Table 9.

Primary tumors in the right colon (ascending colon and cecum) are associated with the worst prognosis.

An increase of 100 units in the LDH value is associated with a 4% increase in hazard of death (Table 10) and an increase of 100 units in the CEA value is associated with a 1% increase in hazard of death (Table 11). Tumor grade, the value of the alkaline phosphatase, and CA 19-9 (Table 12) did not influence prognosis.

## 4. Discussion

Colorectal cancer (CRC) remains to be a significant public health challenge globally, with varying incidences and mortality rates across different regions. CRC incidence rates are highest in Australia/New Zealand and European regions (40.6 per 100,000, males) and lowest in several African regions and Southern Asia (4.4 per 100,000, females). The higher rates observed in European regions are due to lifestyle factors and early detection through screening programs. Eastern Europe exhibits higher mortality rates, especially in males (20.2 per 100,000, males), while Southern Asia exhibits the lowest mortality rate (2.5 per 100,000, females [1,2]. Therefore, the burden of CRC is substantial, requiring comprehensive research to understand its characteristics, and therefore improve patient outcomes. Romania, an Eastern European country with a population of 19.89 million in 2023, lacks a functional cancer registry, which has resulted in limited statistics regarding CRC incidence and mortality. In addition, real-world studies from Romania are scarce. Our investigation explored potential correlations between demographic, clinical, and paraclinical variables and the outcomes in metastatic CRC patients. Notably, we found that 39.11% of our patients had KRAS mutations, consistent with the literature indicating KRAS mutation prevalence in approximately 40% of CRC cases [4].

Our study cohort was evenly distributed between two arms based on KRAS status, and we observed no significant differences between the two groups in terms of sex, age, background, education level, TNM stage, primary tumor localization, or tumor grade [10,11], but our results may be biased by the lack of earlier stage cases. However, we did note a significant difference in the incidence of diabetes mellitus, with 18% of patients in the KRAS wild-type group and only 8% of patients in the KRAS-mutant group having diabetes. This finding is relevant considering emerging evidence that suggests a potential link between KRAS mutation and metformin sensitivity in mCRC, which may impact survival outcome. There is increasing evidence that KRAS mutation determines metformin sensitivity in mCRC by intracellular accumulation through silencing MATE1 (multidrug and toxin extrusion protein 1), with the median overall survival time for patients with diabetes on metformin treatment being 17.5 months longer than that of mCRC patients without diabetes [12]. Unfortunately, the number of patients included in our study is not large enough to enable an analysis of survival for patients on different antidiabetic treatments versus non-diabetic patients. However, this can be explored in larger cohorts, as there is also increasing evidence among other types of KRAS-mutant cancer that metformin can influence survival and its efficacy needs further clinical trials in order to find a place in the treatment continuum of mCRC [13,14,15,16].

Although the observed difference did not reach statistical significance, there was a slight variation in the family history of cancer. A higher proportion of patients in the KRAS-mutant group (5.7%) had a family history of cancer compared to the KRAS wild-type group (2.9%), where at least one case of cancer was reported in the family. This observation aligns with previous studies that explored KRAS mutations in colorectal cancer, indicating a possible association with hereditary non-polyposis colorectal cancer (HNPCC). Hereditary non-polyposis colorectal cancer accounts for ~1–8% of the total colorectal cancer cases on the basis of clinical criteria [17,18,19]. A study conducted by Carla Oliveira on a population of 158 HNPCC patients was able to find a higher frequency of KRAS mutations in HNPCC tumors (40%) as compared with sporadic CRCs (32%), although this difference did not reach statistical significance [20].

The overall survival for the entire study population was 29 months, consistent with data from previous studies for the years 2015–2020. A retrospective review of 1420 patients with de novo metastatic CRC who received their primary treatment at the University of Texas M.D. Anderson Cancer Center had a median OS of 28.8 months for those diagnosed from 2013 to 2015 and 32.4 months for those diagnosed between 2016 and 2019, when new treatment options became available [21]. According to the same study, patients with KRAS-mutant tumors had worse survival relative to the KRAS wild-type patients (median OS of 26.8 vs. 37.1 months, HR = 1.3, *p*-value = 0.0007), a tendency that was also observed in our groups with the KRAS wild-type group having a median overall survival of 31 months compared with the KRAS-mutant group with a median OS of 26 months, but without statistical significance [21]. These findings are consistent with the trend seen in the United States national SEER database [22].

Our study confirmed that education level influenced survival, an observation that has already been validated in multiple populations around the world [23,24,25]. However, as pointed out by Valiati, low levels of education may include many factors that affect survival, like worse access to general health providers, as well as worse nutrition and socioeconomical levels [26]. Pointing out the exact contribution of these features is challenging, but it is important and may be explored in future studies in order to identity groups at high risk of death from cancer and to allow targeted interventions, especially in countries with limited resources where screening programs and access to treatment are limited.

From the perspective of the tumor, T stage, N stage, M stage, and tumor primary site are viewed as predictive factors, and our study confirmed previous reports that have proven that the higher T and N stages are associated with worst prognosis [27,28,29,30,31,32].

Also, primary tumors in the right colon have a dismal prognosis, due to different anatomical and clinical presentations, with more patients being diagnosed in advanced stages due to the late onset of symptoms. Additionally, studies have shown that different sites of colon cancer have differences in disease biology, such as microsatellite instability and differences in gene expression that impact the way the disease reacts to treatment [33,34,35,36,37].

The preoperative CEA level has been confirmed as a prognostic indicator. It is used routinely in clinical practice in the metastatic setting to help monitor treatment response, and there is growing evidence that shows that an increased level of CEA correlates with the CEA metastatic potential, an association confirmed also in our study [38,39,40,41,42].

A metanalysis conducted by Guanghua and his team proved that there is evidence that high lactate dehydrogenase levels indicate poor prognosis among CRC patients, an observation that was also confirmed in the population we analyzed, with an increase of 100 units in the LDH value associated with a 4% increase in hazard of death [43].

Being a retrospective, single-institutional study, our research has inherent limitations. The absence of a national cancer registry in Romania limited our access to comprehensive incidence and mortality data. With regards to the retrospective collection of data, only patients who received their chemotherapy at the Coltea Clinical Hospital were included in the study, since the documentation for many patients who were seen only as consults or second opinions was incomplete. The Coltea Clinical Hospital is a tertiary referral center with 30% of its patients traveling from rural areas around Bucharest for treatment, which tends to skew the patient population to higher socioeconomic status, better performance status, and younger age relative to the broader metastatic CRC population in Romania. These data are relevant to so-called “real-world” metastatic CRC patients in both rural and urban regions, and we hope that further studies will help to gain more insight on disparities of cancer in Romania, allowing decision makers to implement actions adjusted to the different needs of the population with metastatic colorectal cancer.

## 5. Conclusions

This study’s findings align with the global literature, indicating that the prognoses of patients diagnosed with metastatic CRC in Romania exhibit similar characteristics. Despite notable improvements over the past two decades, the five-year survival rate remains relatively low for most patients, emphasizing the urgent necessity for ongoing research to develop more effective treatments for metastatic CRC which is still a deadly disease.

Furthermore, the importance of gathering “real-world” data cannot be overstated, as it enables a deeper understanding of the unique features of metastatic colorectal cancer in diverse populations. By identifying specific groups of patients with poor prognoses, targeted interventions can be implemented to enhance their overall survival rates. Continued efforts in research and data collection are vital to making substantial progress in combating this challenging condition.

## Figures and Tables

**Table 1:** The comparative analysis of demographic characteristics.

Variable	KRAS Wild Type (*N* = 137)	KRAS Mutant (*N* = 88)	*p* Value
Sex, *n* (%)			0.84
Female	61 (45)	38 (43)	
Male	76 (55)	50 (57)	
Age, Mean (SD)	64.71 (9.58)	64.97 (10.33)	0.85
Background, *n* (%)			
Rural	41 (30)	19 (22)	
Urban	96 (70)	69 (78)	
Education level, *n* (%)			0.54
I	42 (31)	21 (24)	
II	59 (43)	41 (47)	
III	36 (26)	26 (30)	
Family history of CRC, *n* (%)	4 (2.9)	5 (5.7)	0.32
Alcohol abuse, *n* (%)	5 (11)	11 (12)	0.72
Obesity, *n* (%)	28 (20)	17 (19)	0.84
Hypertension, *n* (%)	55 (40)	38 (43)	0.65
Diabetes, *n* (%)	25 (18)	7 (8)	0.031
T stage, n (%)			0.71
T1	2 (1.5)	3 (3.4)	
T2	10 (7.3)	5 (5.7)	
T3	82 (60)	50 (57)	
T4	43(31)	30 (34)	
N stage, *n* (%)			0.31
N0	32 (23)	16 (18)	
N1	53 (39)	43 (49)	
N2	52 (38)	29 (33)	
M1 stage at diagnosis, *n* (%)	89 (65)	64 (73)	0.22
Primary tumor			
Rectum, *n* (%)	57 (42)	38 (43)	0.82
Cecum, *n* (%)	22 (16)	11 (12)	0.46
Ascending colon, *n* (%)	3 (2.2)	4 (4.5)	0.44
Transverse colon, *n* (%)	5 (3.6)	6 (6.6)	0.35
Rectosigmoid junction, *n* (%)	17 (12)	12 (14)	0.79
Sigmoid colon, *n* (%)	33 (24)	17 (19)	0.40
Grade of differentiation, *n* (%)			0.89
G1	18 (13)	11 (12)	
G2	91 (66)	61 (69)	
G3	28 (20)	16 (18)	

**Table 2:** The Cox regression for the KRAS wild-type and KRAS-mutant groups.

Predictor	N	Deaths	HR (95% CI)	*p* Value
KRAS status				
Wild type	137	126		
Mutant	88	80	0.95 (0.72 to 1.26)	0.726

HR, hazard ratio; CI, confidence interval.

**Table 3:** Overall survival according to the level of education Cox regression.

Predictor	N	Deaths	HR (95% CI)	*p* Value
Level of education				
I	63	62	-	
II	100	89	0.67 (0.48 to 0.93)	0.017
III	62	55	0.70 (0.48 to 1.00)	0.053

HR, hazard ratio; CI, confidence interval.

**Table 4:** Overall survival according to T stage.

Strata T Stage	Deaths (%)	RMST	Survival Median (95% CI)
T1	3/5 (60.00)	55.80	62.00 (29 to N/A)
T2	14/15 (93.33)	50.50	35.00 (12.00 to 54.00)
T3	118/132 (89.39)	42.50	32.00 (27.00 to 40.00)
T4	71/73 (97.26)	26.30	24.00 (20.00 to 28.00)

N/A, not applicable.

**Table 5:** Overall survival according to T stage Cox regression.

Predictor	N	Deaths	HR (95% CI)	*p* Value
T stage				
T1	5	3	-	
T2	15	14	2.02 (0.57 to 7.15)	0.275
T3	132	118	2.08 (0.66 to 6.57)	0.210
T4	73	71	3.96 (1.24 to 12.6)	0.020

HR, hazard ratio; CI, confidence interval.

**Table 6:** Overall survival according to N stage.

Strata N Stage	Deaths (%)	RMST	Survival Median (95% CI)
N0	42/48 (87.50)	53.40	37.50 (32.00 to 57.00)
N1	86/96 (89.58)	41.90	31.00 (24.00 to 39.00)
N2	78/81 (96.29)	24.00	30.00 (20.00 to 29.00)

**Table 7:** Overall survival according to N stage Cox regression.

Predictor	N	Deaths	HR (95% CI)	*p* Value
N stage				
N0	48	42	-	
N1	96	86	1.53 (1.05 to 2.23)	0.027
N2	81	78	2.40 (1.61 to 3.56)	<0.001

HR, hazard ratio; CI, confidence interval.

**Table 8:** Overall survival according to M stage at diagnosis.

Strata M Stage	Deaths (%)	RMST	Survival Median (95% CI)
M0	65/72 (90.27)	51.10	39.00 (32.00 to 50.00)
M1	141/153 (92.15)	31.80	25.00 (23.00 to 30.00)

**Table 9:** Overall survival according to the primary tumor location.

Predictor	N	Deaths	HR (95% CI)	*p* Value
Rectum				
No	130	121	-	
Yes	95	85	4.18 (0.84 to 20.9)	0.081
Cecum				
No	192	173	-	
Yes	33	33	5.19 (1.00 to 26.9)	0.050
Ascending colon				
No	218	200	-	
Yes	7	6	4.70 (0.78 to 28.4)	0.091
Transverse colon				
No	214	196	-	
Yes	11	10	3.54 (0.63 to 19.8)	0.15
Rectosigmoid				
No	196	180	-	
Yes	29	26	4.43 (0.89 to 22.1)	0.069
Sigmoid				
No	175	160	-	
Yes	50	46	3.53 (0.69 t0 18.0)	0.13

HR, hazard ratio; CI, confidence interval.

**Table 10:** Overall survival according to the LDH increased levels Cox regression.

Predictor	N	Deaths	HR (95% CI)	*p* Value
LDH/100	225	206	1.04 (1.02 to 1.06)	<0.001

HR, hazard ratio; CI, confidence interval.

**Table 11:** Overall survival according to the CEA increased levels Cox regression.

Predictor	N	Deaths	HR (95% CI)	*p* Value
CEA/100	225	206	1.01 (1.00 to 1.03)	0.068

HR, hazard ratio; CI, confidence interval.

**Table 12:** Overall survival according to the CA 19-9 increased levels Cox regression.

Predictor	N	Deaths	HR (95% CI)	*p* Value
CA 19-9	225	206	1.00 (0.99 to 1.01)	0.200

HR, hazard ratio; CI, confidence interval.

## Data Availability

Data are available on request due to ethical restrictions. The data presented in this study are available on request from the corresponding author. The data are not publicly available due to the policy of the Coltea Clinical Hospital to have the approval of the Ethics Committee for each new research study.
